# Association of Balance Scores With Cognitive Function in Older Adults

**DOI:** 10.1093/geroni/igaa057.504

**Published:** 2020-12-16

**Authors:** Claire Meunier, Ellen Smit, Annette Fitzpatrick, Michelle Odden

**Affiliations:** 1 Oregon State University, Corvallis, Oregon, United States; 2 University of Washington, Seattle, Washington, United States; 3 Stanford University, STANFORD, California, United States

## Abstract

Previous research has reported an association between balance and cognitive function; however, there is a paucity of data on this relationship over time. This study examined the cross-sectional and longitudinal association between balance and cognitive function in 4,811 participants aged 65 years and older in the Cardiovascular Health Study (CHS). Cognitive function measures included the Modified Mini-Mental State Examination (3MSE) and Digit Symbol Substitution Test (DSST); measures were collected annually for six years, starting in 1992-1993. A tandem stance balance test was administered at baseline; this test was held for 5 seconds. Cross-sectional and longitudinal models were adjusted for demographics, behavioral and disease covariates. We found that participants with worse balance scores had lower cognitive function scores, and this effect was limited to participant who were above the median age (76 years) (p-value for interaction = 0.03 in a demographic-adjusted model). Participants 76 years and older who failed the balance test had an average adjusted decline of -0.97 (95% CI: 1.20, 2.29) 3MSE scores per year more than participants who completed the balance test. DSST showed similar results; participants with poor balance decreased -0.21 (95% CI: -0.37, -0.05) points per year more than participants who completed the balance test. The adjusted Cox proportional hazard model found participants with poorer balance had a higher risk of cognitive impairment over the six years (HR= 1.72 95% CI: 1.30, 2.29). A better understanding of the pathophysiological link between balance and cognition may inform strategies to prevent cognitive decline.

